# Unexpected fatal hemodynamic collapse during MRI anaesthesia in an 11-month-old infant with suspected neuroblastoma: a case report

**DOI:** 10.1186/s12887-026-06826-2

**Published:** 2026-04-07

**Authors:** Peter Bakkegaard, Peter Søndergaard Thyrrestrup, Christina Friis Jensen

**Affiliations:** 1 Department of Anaesthesiology and Intensive Care, Aalborg University Hospital, Aalborg, Denmark; 2 Department of Paediatrics, Aalborg University Hospital, Aalborg, Denmark

**Keywords:** Catecholamine-secreting neuroblastoma, Hypertensive crisis, General anaesthesia, Infant, Hemodynamic collapse

## Abstract

**Background:**

Neuroblastoma is the most common extracranial solid tumours in children, but catecholamine-secreting variants are rare. Unlike pheochromocytoma, routine preoperative α-blockade and extended perioperative monitoring are not recommended for asymptomatic neuroblastoma, as clinically significant catecholamine secretion is considered exceptional. We present a fatal case of hemodynamic collapse during general anaesthesia for magnetic resonance imaging in an infant with undiagnosed catecholamine-secreting neuroblastoma. Subtle preoperative symptoms were present, the risk was underestimated, and lack of preparedness contributed to the outcome.

**Case presentation:**

An 11-month-old boy was admitted with failure to thrive, excessive thirst, nocturnal polyuria, and episodes of sweating. He appeared clinically stable except for hypertension (114/91 mmHg) and tachycardia 144 bpm, attributed to agitation. He was underweight (weight 8.1 kg, 3rd percentile) with suspected hepatomegaly. Ultrasound revealed a large abdominal mass suspicious for hepatoblastoma or neuroblastoma. Laboratory values at admittance were normal.

The following day, magnetic resonance imaging under general anaesthesia was scheduled. Induction with sevoflurane followed by conversion to total intravenous anaesthesia and intubation was uneventful, but the patient remained tachycardic (150–160 bpm) and hypertensive (151/115 mmHg). Despite fluid bolus, increased anaesthetic depth, and manual tumours displacement tachycardia worsened (up to 240 bpm), prompting abortion of the scan. He subsequently developed hypotension, mottling and cardiac arrest. Resuscitation was unsuccessful. Urinary catecholamine metabolites had been sampled before induction of anaesthesia, but the markedly elevated results were only available after the fatal event (vanillylmandelate 900 µmol/L (reference <12) and homovanillate 197 µmol/L (reference <21)). Autopsy revealed a large adrenal tumours compressing the inferior vena cava. Histology confirmed poorly differentiated neuroblastoma with neuroendocrine differentiation and extensive metastases.

**Conclusions:**

This case illustrates a catastrophic manifestation of catecholamine-secreting neuroblastoma, in which general anaesthesia alone unmasked a hypertensive crisis and fatal collapse. Subtle preoperative symptoms were present, but the risk was underestimated, and preparedness for a catecholamine crisis was limited. Vigilance for unexplained hypertension, flushing, or sweating in children with suspected neuroblastoma is essential. When catecholamine secretion is suspected, both preventive strategies and prompt pharmacological treatment of hypertensive crises, including α-blockade and titratable vasodilators, should be considered to prevent rare but devastating events.

**Supplementary Information:**

The online version contains supplementary material available at 10.1186/s12887-026-06826-2.

## Background

Neuroblastoma (NBL) is the third most common paediatric malignancy and the most frequent solid extracranial tumour in children, with 8–10 new cases diagnosed annually in Denmark, corresponding to an incidence of approximately 8.55 per million children [1]. NBL arises from neural crest-derived sympathetic precursors and has the potential to synthesize catecholamines. However, most NBLs lack the enzymatic capacity to convert catecholamines into active metabolites, and clinically functional NBL (fNBL) is therefore very rare [2, 3]. Symptoms may include hypertension, tachycardia, flushing, and sweating, but are often absent or subtle.

In children with catecholamine-secreting tumours, anaesthesia and surgical manipulation are recognised triggers of hypertensive crisis [4]. Since clinically significant catecholamine secretion is uncommon in NBL, unlike pheochromocytoma, routine α-blockade and extended perioperative monitoring are not recommended in asymptomatic patients [3, 5].

This case illustrates how subtle, nonspecific preprocedural signs of fNBL were overlooked, resulting in fatal hemodynamic collapse during standard of care general anaesthesia (GA) for diagnostic magnetic resonance imaging (MRI).

## Case presentation

**Fig. 1:**
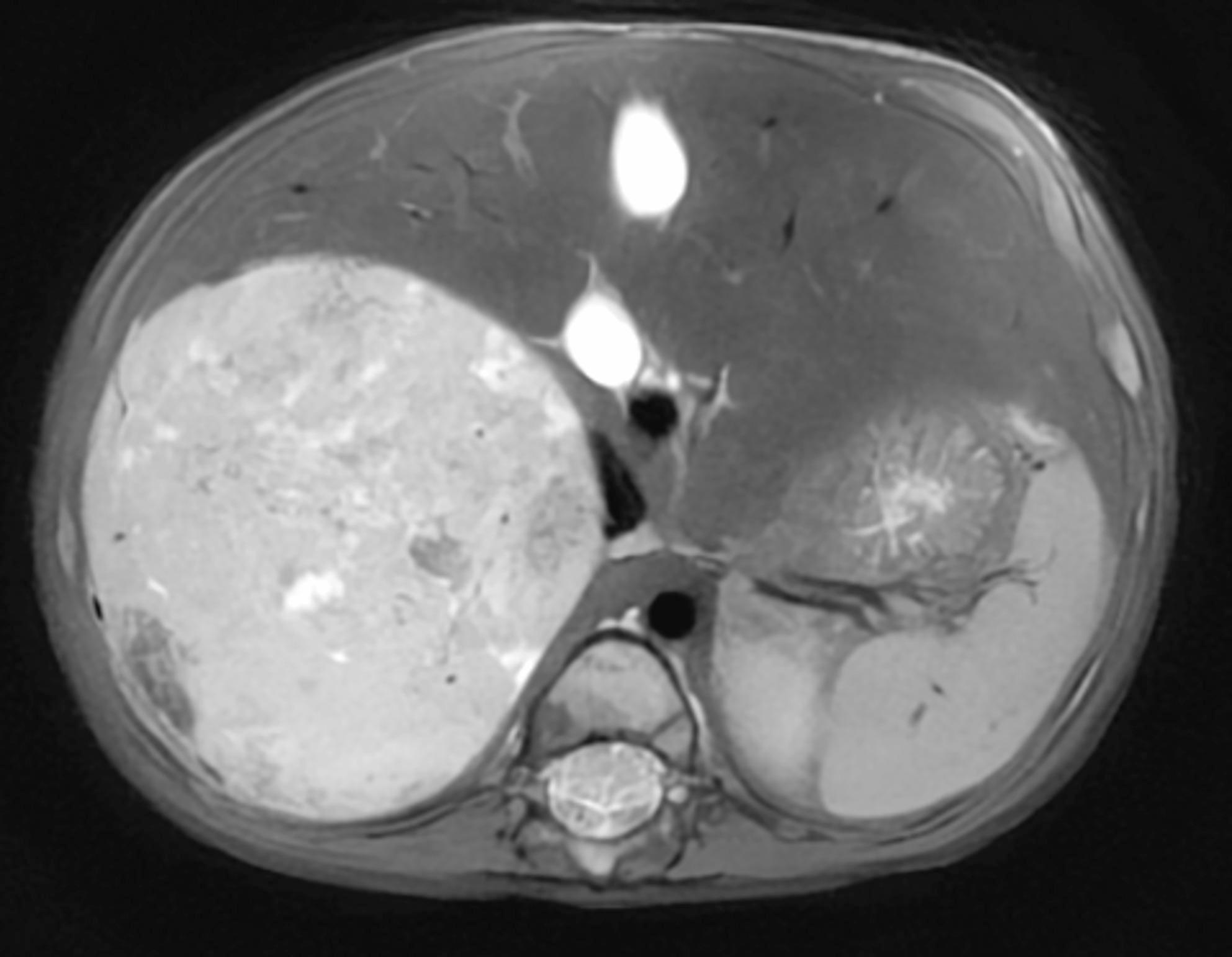
Ultrasound of the liver and biliary system performed on the day of referral, demonstrating an 8 × 7.5 × 8 cm heterogeneous, rounded mass in the right hepatic lobe, suspicious for hepatoblastoma or NBL

An 11-month-old boy was admitted with failure to thrive, excessive thirst and nocturnal polyuria. Episodes of sweating had been present for a long period and he had always been regarded as a “warm child”. On admission he appeared non-acutely ill with normal vital signs, except for a blood pressure of 114/91 mmHg and relative tachycardia 144 bpm. His weight was 8.1 kg (3rd percentile). As the child was agitated and the symptoms had been present for several months, neither the vital signs nor the symptoms raised immediate clinical concern. Abdominal ultrasound showed a heterogeneous mass (8 × 7.5 × 8 cm) suspicious for hepatoblastoma or NBL (Fig. 1). Routine blood tests, including electrolytes, liver and thyroid function, infection markers, and alpha-fetoprotein were normal. Urinary catecholamine metabolites had been sampled prior to induction of anaesthesia, but the markedly elevated results were not available at the time of the MRI procedure and only became known after the fatal event. Vanillylmandelate (VMA) (900 µmol/L, ref < 12) and homovanillate (HVA) (197 µmol/L, ref < 21).

**Fig. 2:**
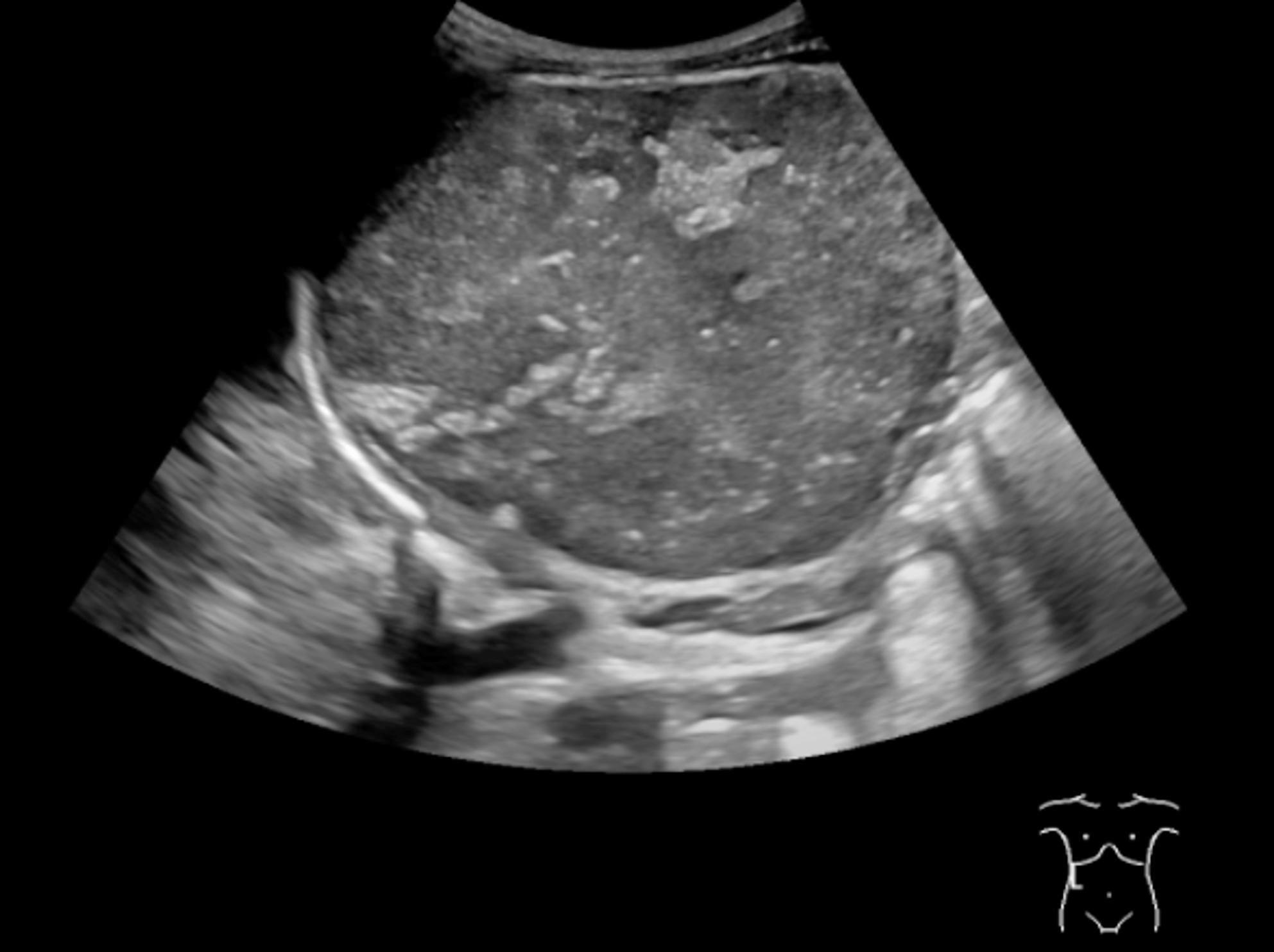
Axial T2-weighted MRI sequence of the abdomen, without contrast agent; slice thickness 3 mm

The next day, diagnostic MRI under GA was planned. Intravenous access proved difficult; anaesthesia was therefore induced with sevoflurane inhalation, then converted to total intravenous anaesthesia (TIVA) with propofol (7 mg·kg^− 1^·h^− 1^) and remifentanil (0.2 µg·kg^− 1^·min^− 1^), and a remifentanil bolus of 4 µg/kg was administered for intubation. Due to difficulty with placement of an IV-line Sevoflurane induction was prolonged for 8–10 min and therefore no initial propofol bolus was given. During attempts at intravenous access and inhalational induction with sevoflurane, the child was seated with a caregiver present. Initially cooperative and curious, he became increasingly agitated during repeated attempts at intravenous cannulation, with crying and resistance. Brief physical restraint around the nose and mouth was required only to ensure a tight-fitting mask during induction. Following induction, vital signs were recorded and the patient was placed in the supine position for the remainder of the procedure. Unexpectedly, the patient remained tachycardic (heart rate 150–160 bpm) and hypertensive (blood pressure 151/115 mmHg). A crystalloid bolus (10 mL/kg Ringer’s acetate) was administered due to concern for relative hypovolemia (tachycardia and difficult IV-line placement). Despite persistent hypertension, repeated propofol boluses of 20–30 mg were administered in attempts to deepen anaesthesia, and manual tumour displacement had little effect. Tachycardia subsequently escalated to 240 bpm, prompting abortion of the scan (Fig. 2). A markedly distended bladder was noted and considered a possible cause of tachycardia and hypertension. Urethral catheterization was unsuccessful, but suprapubic catheterization drained approximately 200 mL of urine, resulting in a partial reduction of heart rate to 190–200 bpm.

Intraoperatively, point-of-care ultrasound (POCUS) was performed to assess cardiac function and volume status in the setting of severe hypertension, tachycardia, and suspected inferior vena cava compression. Image acquisition was limited by pronounced tachycardia; nevertheless, subjective visual assessment suggested hyperdynamic ventricular function and a possible thickening of the left ventricular wall. These findings were subsequently not substantiated, as the autopsy report demonstrated a heart with normal anatomy.

To stabilize the hemodynamics, sevoflurane (minimal alveolar concentration 0.5) was added to TIVA. Suspecting inferior vena cava syndrome, another attempt at manual tumour displacement was made without effect. Attempts were made to establish an arterial line and more advanced IV access but unsuccessful. Due to rapid deterioration of clinical status further attempts were ceased. Shortly thereafter, the patient developed mottling, delayed capillary refill, hypotension, and cardiac arrest. Advanced resuscitation was unsuccessful after 30 min (Table 1).

**Table 1:** Timeline of clinical course, hemodynamics and interventions

Time	Event	HR (bpm)	BP (mmHg)	Interventions	Clinical interpretation
Admission	Initial clinical assessment	144	114/91	–	Unexplained hypertension and tachycardia (early red flag, attributed to agitation)
08:30	Arrival, failed IV attempts	–	–	–	Agitation, sympathetic activation
09:00	Induction (sevoflurane), intubation	–	–	–	Induction stress
09:15	Persistent tachycardia and hypertension	160	151/115	Fluid bolus 10 mL/kg	Misinterpreted as hypovolemia
09:45	MRI start	160	–	–	Ongoing sympathetic activation
10:00	Inadequate depth, escalating instability	↑	↑	Propofol boluses, ↑ infusion	Escalating catecholamine surge
10:20	MRI interrupted	–	–	–	Clinical deterioration
10:45	Marked tachycardia and hypertension	220–240	↑	–	Hypertensive crisis
11:15	Bladder drainage	190–200	–	Suprapubic catheter	Partial transient improvement
11:45	Increased anaesthetic depth and POCUS	↓	↓	Addition of sevoflurane	Reduced venous return and cardiac depression
12:00	Attempts at advanced IV access	↓	↓	-	Decompensation
12:30	Clinical deterioration	–	↓	Fluid bolus	Decompensation
12:50	Cardiac arrest	–	–	CPR	Circulatory collapse

### Post-mortem findings

Autopsy showed a large adrenal tumour compressing the inferior vena cava, with extensive necrosis and metastases to lymph nodes and bone marrow. Histology confirmed poorly differentiated NBL with focal aggressive features and neuroendocrine differentiation. Cardiac histology was largely unremarkable, with normal cardiomyocytes and no fibrosis or storage disease. Focal disarray in the left ventricular anterior wall was assessed as clinically insignificant (Table 1).

## Discussion and conclusion

This tragic case illustrates a rare manifestation of NBL, where GA alone unmasked an undiagnosed catecholamine-producing tumour and triggered fatal hemodynamic collapse. While hypertensive crises have been reported during surgical manipulation of NBL [6], we found no previous cases in which GA for diagnostic imaging alone precipitated such a rapid and irreversible course.

Importantly, the timeline demonstrates that abnormal hemodynamics were already present at admission, preceding anaesthesia, suggesting an underlying catecholamine-driven state rather than an isolated anaesthetic complication. This indicates that early warning signs were present but not recognized, and that the subsequent clinical course reflects a progressive escalation from sympathetic activation to a fulminant catecholamine crisis.

NBL typically presents with nonspecific symptoms such as failure to thrive, pain, fever, weight loss, or an abdominal mass [7]. Because tumour cells usually lack dopamine-β-hydroxylase (DBH) and phenylethanolamine-N-methyltransferase, clinically significant catecholamine secretion is considered rare, with arterial hypertension reported in only 2–19% of patients [3, 7]. In our case, severe hypertension [8] was documented at admission but dismissed as unreliable. Consequently, catecholamine secretion was not suspected, and a standard anaesthetic protocol was applied, consistent with the current recommendations for asymptomatic children with suspected NBL [3, 5]. In retrospect, the narrow pulse pressure combined with tachycardia at admission may have reflected increased systemic vascular resistance due to activation of the renin-angiotensin-aldosterone system, release of neurohumoral mediators such as vasopressin, and possible compression of the renal arteries, in addition to relative hypovolaemia and impaired venous return. Such findings should raise concern for compromised preload, and underlying cardiovascular stress. POCUS was not performed pre-induction but might have provided additional information regarding cardiac function and volume status and should be considered in similar cases with unexplained hypertension and tachycardia. The ensuing hemodynamic destabilization was therefore unexpected, and no specific pharmacological strategy for a catecholamine crisis was in place.

The tachycardia and difficult IV-line placement were interpreted as signs of relative hypovolemia. In retrospect, the decision to administer an initial fluid bolus may reflect heuristic-driven reasoning, with tachycardia attributed to hypovolemia rather than recognition of a pathological hypertensive state related to catecholamine excess. This illustrates how cognitive bias and anchoring to more common peri-anaesthetic causes of tachycardia may delay recognition of a catecholamine crisis in rare but high-risk situations.

Although agents classically associated with hypertensive crises were not administered, the potential role of propofol deserves consideration. No initial propofol bolus was given; however, repeated boluses were administered during the procedure in attempts to deepen anaesthesia. Propofol is generally considered a safe agent in patients with catecholamine-secreting tumours, as it typically reduces systemic vascular resistance, myocardial contractility, and baroreceptor reflex activity, and has not been regarded as a precipitating factor in previous reports [9, 10]. Nevertheless, a contributory role cannot be completely excluded, as an abrupt reduction in systemic vascular resistance may theoretically provoke reflex sympathetic activation in the presence of an active catecholamine-secreting tumour. Similar theoretical considerations may also apply to sevoflurane, which shares several effects on systemic vascular resistance and myocardial function. We therefore consider the catecholamine surge in this case to be the result of multiple converging mechanisms, including mechanical, physiological, and pharmacological factors.

Catecholamine-induced Takotsubo-like cardiomyopathy has recently been described in children with catecholamine-secreting tumours, particularly pheochromocytoma [11]. However, this mechanism appears unlikely in the present case, as POCUS suggested preserved to hyperdynamic ventricular function without regional wall motion abnormalities, and post-mortem examination demonstrated normal myocardial architecture. Accordingly, the subsequent hypotension and cardiac arrest were most likely multifactorial. A massive catecholamine surge could have caused an extreme and abrupt increase in afterload with secondary acute myocardial dysfunction and impaired cardiac output. In addition, direct catecholamine-mediated myocardial toxicity may have contributed. Despite attempts at manual tumour displacement, haemodynamic compromise may have been further exacerbated by reduced preload and increased afterload due to compression of the inferior vena cava and the aorta, by the large abdominal tumour, compounded by general anaesthesia and the supine position. In similar situations, repositioning to a left lateral or prone position may be considered to alleviate mechanical vascular compression. Together, these mechanisms are consistent with a rapidly evolving circulatory collapse rather than isolated primary cardiac failure.

Besides increasing anaesthetic depth (Propofol 7–10 mg·kg⁻¹·h⁻¹ and remifentanil 0.2–1.0 µg·kg⁻¹·min) no specific antihypertensive treatment was initiated. In retrospect, the combination of severe hypertension and extreme tachycardia represented a hypertensive emergency requiring immediate pharmacological treatment. Acute stabilization with rapidly titratable vasodilators (e.g., sodium nitroprusside or nitroglycerin) and, if needed, short-acting beta-blockade *after* adequate vasodilation, might have mitigated the crisis [12]. In cases where catecholamine secretion is suspected α-blockade should be considered prior to any subsequent anaesthetic exposure [4, 12, 13].

Hypertensive crises in NBL have been linked to nociceptive stimuli (e.g., intubation) and anaesthetic agents with sympathomimetic or histamine-releasing properties. In known NBL, intraoperative hypertension occurs in approximately 13% of cases [14]. Although no such agents were used in this case, intubation and invasive procedures (e.g., catheter placement, manual tumour displacement) may have contributed to the crisis. Sedative premedication might have attenuated the stress response, but no guidelines exist for the preoperative management of children with suspected NBL.

In Denmark, paediatric anaesthesia is traditionally conducted in a calm and reassuring manner to promote the child’s comfort and minimize pharmacological premedication. In this case, the child was markedly agitated, and postponement of anaesthesia with administration of sedative premedication (e.g., oral clonidine) to achieve sufficient effect was considered but ultimately not pursued for logistical reasons. The potential use of intraoperative sympatholytic agents such as dexmedetomidine or clonidine was not considered. While such agents may attenuate stress-related sympathetic activation and tachycardia, they do not replace specific α-blockade and evidence for their preventive role in catecholamine-secreting tumours is limited. Nevertheless, in retrospect, premedication and early sympatholytic strategies might have reduced the peri-induction stress response in this highly vulnerable patient.

The tumour was classified as poorly differentiated, stage 4 NBL with extensive metastases to bone marrow and lymph nodes, without MYCN amplification, consistent with intermediate risk [7]. Nevertheless, it exhibited highly pathological features, including bizarre multinucleated giant cells, previously associated with unfavourable histology and rapid progression [15]. These features may have contributed to the fulminant clinical deterioration despite intermediate-risk biology.

Catecholamine secretion was not suspected before MRI, as urinary VMA/HVA – although positive in most NBL – does not reliably distinguish functional tumours [16]. Previous reports describe hypertensive episodes during anaesthesia for imaging, but these were managed successfully by deepening anaesthesia [5, 14]. Seefelder et al. [13] reported hypertensive crisis after intubation in a child with known catecholamine-producing NBL who had received preoperative antihypertensive therapy, underscoring the importance of recognising symptoms in advance [4, 13, 17].

In our case, subtle preoperative signs (flushing, sweating, hypertension) were misinterpreted, and catecholamine secretion was not suspected. Consequently, no specific preoperative measures such as α-blockade, enhanced monitoring, or multidisciplinary planning were implemented.

While biochemical confirmation may support the diagnosis, clinical risk assessment should not rely solely on laboratory results. The presence of unexplained hypertension, tachycardia, or sweating in a child with suspected neuroblastoma should raise suspicion of catecholamine activity and prompt precautionary perioperative planning, regardless of biochemical confirmation.

In similar clinical scenarios, three key considerations may improve patient safety:

1. Recognition of red flags, including unexplained hypertension, tachycardia, flushing, or sweating.
2. Consideration of postponing non-urgent anaesthesia until further risk assessment is completed.
3. If anaesthesia is required, preparation for a potential catecholamine crisis, including availability of titratable vasodilators, invasive monitoring, and multidisciplinary planning.

Such an approach may help mitigate the risk of rare but life-threatening perioperative complications.

In summary, this case highlights the difficulty of identifying the rare subset of NBL patients with clinically significant catecholamine secretion. For anaesthesiologists and paediatricians, vigilance for unexplained hypertension, tachycardia, flushing, or sweating is essential. When fNBL is suspected both preoperative strategies (α-blockade, invasive monitoring, postoperative intensive care) and prompt pharmacological treatment of hypertensive crises are crucial to prevent catastrophic outcomes. Increased awareness and systematic risk assessment may help to avoid similar fatal events.

## Learning points

- Catecholamine secretion in neuroblastoma (NBL) is rare but can have catastrophic consequences.
- General anaesthesia alone, without surgical manipulation, may unmask a hypertensive crisis in functional NBL.
- Subtle clinical signs such as flushing, unexplained sweating, or hypertension should raise suspicion of catecholamine secretion.
- In at-risk children, preoperative α-blockade, invasive monitoring, and postoperative intensive care may help prevent catastrophic outcomes.
- Severe hypertension and tachycardia during anaesthesia constitute a medical emergency requiring immediate pharmacological treatment in addition to preventive strategies.

## Supplementary Information

Supplementary Material 1.

## Data Availability

No datasets were generated or analysed during the current study.

## Abbreviations

DBH: Dopamine-β-hydroxylase
fNBL: Functional/catecholamine secreting neuroblastoma
HVA: Urine homovanillate
MRI: Magnetic Resonance Imaging
NBL: Neuroblastoma
VMA: Urine vanillylmandelate
