# Supplementary material for: Unexpected fatal hemodynamic collapse during MRI anaesthesia in an 11-month-old infant with suspected neuroblastoma: a case report
Source: BMC Pediatr. 2026 Apr 7;26:455. doi: 10.1186/s12887-026-06826-2 (PMC13192122; doi:10.1186/s12887-026-06826-2)
Supplement: Supplementary file 1 — Supplementary Material 1. [file 12887_2026_6826_MOESM1_ESM.docx]

CARE Checklist – Draft for Submission

**1. Title**

*Unexpected Fatal Hemodynamic Collapse During MRI Anaesthesia in an 11-Month-Old Infant With Suspected Neuroblastoma: A Case Report*

­­

**2. Key Words**

Catecholamine-secreting neuroblastoma; Hypertensive crisis; General anaesthesia; Infant; Hemodynamic collapse

­­­

**3. Abstract**

Structured abstract with Background, Case presentation, and Conclusions. Length: 350 words. Describes the clinical problem, key diagnostic findings, anaesthetic course, autopsy results, and major learning points.

**4. Introduction**

Background on neuroblastoma as the most common extracranial solid tumour in children. Functional/catecholamine-secreting variants are rare, unlike pheochromocytoma. Rationale: to highlight a unique case where GA alone during MRI triggered fatal collapse, underscoring the need for vigilance and preparedness.

**5. Patient Information**

- 11-month-old male infant.
- Clinical symptoms: failure to thrive, excessive thirst, nocturnal polyuria, episodes of flushing and sweating.
- No relevant past medical history.
- No family history of malignancy or endocrine disease reported.

**6. Clinical Findings**

- Underweight with marked hepatomegaly.
- Blood pressure: 114/91 mmHg (above 99th percentile + 5 mmHg).
- Heart rate: 144 bpm.
- Otherwise clinically stable on admission.

**7. Timeline**

- Day 0 (Admission): Presentation with weight loss, polyuria, flushing, sweating. Hypertension noted. Ultrasound: large abdominal mass. Blood tests: normal. Urinary catecholamines ordered.
- Day 1: MRI under GA. Anaesthesia with sevoflurane → TIVA (propofol, remifentanil). Persistent tachycardia and hypertension. Crisis developed → cardiac arrest → unsuccessful resuscitation.
- Day 2+: Post-mortem examination confirmed catecholamine-secreting, poorly differentiated neuroblastoma with metastases.

**8. Diagnostic Assessment**

- Ultrasound: heterogeneous 8 × 7.5 × 8 cm abdominal mass (suspected hepatoblastoma vs NBL).
- Laboratory: initially normal. Later markedly elevated urinary VMA (900 µmol/L; ref <12) and HVA (197 µmol/L; ref <21).
- Autopsy: adrenal tumour compressing vena cava, poorly differentiated neuroblastoma, neuroendocrine differentiation, metastases to lymph nodes and bone marrow, multinucleated giant cells.
- No genetic MYCN amplification.

**9. Therapeutic Intervention**

- GA induction: sevoflurane due to difficult IV access.
- Conversion to TIVA (propofol, remifentanil).
- Supportive measures: crystalloid bolus, increased anaesthetic depth, manual tumour displacement, catheterisation.
- ALS performed after cardiac arrest.

**10. Follow-up and Outcomes**

- Fatal outcome despite 30 minutes of advanced resuscitation.

**11. Discussion**

- GA alone unmasked a hypertensive crisis in a previously undiagnosed catecholamine-secreting neuroblastoma.
- Unlike previous reports, no surgical manipulation was performed.
- Hypertension at admission was underestimated.
- Histology revealed poorly differentiated NBL with multinucleated giant cells, features associated with aggressive tumour behaviour.
- Learning point: vigilance for catecholamine secretion is essential when hypertension, flushing, or sweating are present, even in seemingly stable NBL patients.

**12. Patient Perspective**

Not applicable due to patient’s age and fatal outcome.

**13. Informed Consent**

Written informed consent for publication, including clinical details and images, was obtained from the patient’s parents.
